# Supplementary material for: The Effect of Transcranial Electrical Stimulation on the Recovery of Sleep Quality after Sleep Deprivation Based on an EEG Analysis
Source: Brain Sci. 2023 Jun 9;13(6):933. doi: 10.3390/brainsci13060933 (PMC10295838; doi:10.3390/brainsci13060933)
Supplement: Supplementary file 1 [file brainsci-13-00933-s001.zip › brainsci-2383199-supplementary.pdf]

Table S1 face,(mean±sd)

	SD36h		RS	
Face expression	tDCS	BC	tDCS	BC
neutral	0.19±0.21	0.32±0.28	0.39±0.33	0.38±0.27
happy	0.06±0.11	0.01±0.02	0.06±0.16	0.05±0.10
sad	0.25±0.35	0.24±0.35	0.22±0.32	0.06±0.11
angry	0.11±0.25	0.17±0.24	0.12±0.22	0.23±0.31
surprised	0.32±0.35	0.19±0.29	0.19±0.32	0.22±0.32
scared	0.04±0.11	0.04±0.13	0.00±0.00	0.02±0.04
disgusted	0.13±0.22	0.03±0.07	0.04±0.10	0.03±0.06

Table S2 Result of Two factor analysis of facial expression. *** p < 0.01

emotion	effect	Estimate	Std.err	Wald	P
Angry	group	0.06	0.08	0.63	0.426
	session	0.02	0.05	0.10	0.756
	Session*group	0.04	0.08	0.30	0.583
Disgusted	group	-0.11	0.06	3.71	0.054
	session	-0.10	0.04	5.59	0.018***
	session*group	0.10	0.05	4.49	0.034***
Happy	group	-0.05	0.03	3.60	0.058
	session	-0.01	0.05	0.01	0.922
	session*group	0.04	0.05	0.69	0.408
Neutral	group	0.14	0.08	2.66	0.103
	session	0.20	0.07	7.78	0.005***
	session*group	-0.15	0.11	1.83	0.177
Sad	group	-0.01	0.12	0.01	0.942
	session	-0.04	0.06	0.32	0.570
	session*group	-0.15	0.11	1.89	0.169
Scared	group	0.00	0.04	0.00	0.967
	session	-0.04	0.03	2.09	0.148
	session*group	0.02	0.04	0.23	0.630
Surprised	group	-0.13	0.11	1.36	0.244
	session	-0.13	0.09	2.11	0.147
	session*group	0.16	0.13	1.59	0.207

Table S3. The results of two-factor analysis of variance

Channels	alpha						beta					
	group		session		Interaction effect		group		session		Interaction effect	
	F	P'	F	P'	F	P'	F	P'	F	P'	F	P'
Fpz	0.04	0.993	21.59	0.000	1.68	0.294	0.34	0.674	22.35	0.000	2.86	0.205
Fp1	0.01	0.993	38.09	0.000	3.48	0.192	0.67	0.591	28.70	0.000	7.86	0.042
Fp2	0.20	0.993	16.79	0.000	3.03	0.192	2.80	0.463	13.74	0.001	3.48	0.168
AF3	0.03	0.993	34.24	0.000	4.72	0.169	0.59	0.602	67.22	0.000	14.42	0.008
AF4	0.00	0.993	28.48	0.000	4.42	0.171	0.32	0.674	46.42	0.000	8.17	0.042
AF7	0.56	0.993	40.29	0.000	3.05	0.192	1.43	0.507	18.31	0.000	3.69	0.163
AF8	0.87	0.993	22.00	0.000	2.50	0.221	3.32	0.418	21.54	0.000	3.78	0.163
Fz	0.05	0.993	25.60	0.000	3.09	0.192	1.46	0.507	62.16	0.000	11.08	0.019
F1	0.54	0.993	36.50	0.000	7.33	0.106	2.34	0.485	63.78	0.000	17.30	0.007
F2	0.00	0.993	28.46	0.000	4.80	0.169	0.76	0.587	62.55	0.000	16.39	0.007
F3	1.36	0.993	55.62	0.000	9.18	0.076	1.97	0.485	67.13	0.000	13.77	0.008
F4	0.50	0.993	35.08	0.000	6.35	0.125	0.62	0.601	48.16	0.000	10.17	0.024
F5	0.41	0.993	45.62	0.000	6.72	0.120	0.04	0.875	35.16	0.000	9.80	0.024
F6	1.27	0.993	27.22	0.000	1.06	0.374	0.87	0.587	33.23	0.000	0.27	0.748
F7	0.90	0.993	35.08	0.000	10.02	0.076	0.31	0.674	20.88	0.000	7.94	0.042
F8	1.24	0.993	24.28	0.000	0.61	0.493	0.95	0.587	20.73	0.000	0.13	0.782
FCz	0.16	0.993	16.22	0.000	1.40	0.329	2.58	0.463	31.77	0.000	4.12	0.143
FC1	1.42	0.993	22.26	0.000	3.59	0.192	5.30	0.356	32.91	0.000	5.01	0.112
FC2	0.88	0.993	21.28	0.000	2.66	0.216	4.57	0.356	31.42	0.000	5.83	0.085
FC3	5.29	0.831	40.68	0.000	13.29	0.055	7.97	0.356	64.48	0.000	15.98	0.007
FC4	1.91	0.993	26.51	0.000	1.58	0.306	4.58	0.356	26.11	0.000	2.48	0.236
FC5	1.26	0.993	15.05	0.001	3.11	0.192	0.70	0.587	7.45	0.014	1.35	0.348
FC6	1.96	0.993	24.36	0.000	1.22	0.348	1.16	0.553	24.92	0.000	0.18	0.782
FT7	3.59	0.993	19.72	0.000	9.03	0.076	2.05	0.485	12.39	0.002	4.32	0.137
FT8	6.43	0.831	44.77	0.000	5.20	0.169	4.43	0.356	18.72	0.000	1.48	0.333
Cz	0.21	0.993	0.65	0.427	1.31	0.341	0.27	0.674	1.38	0.253	1.72	0.293
C1	0.06	0.993	3.14	0.092	0.04	0.863	1.82	0.485	6.96	0.017	0.91	0.454
C2	0.10	0.993	7.66	0.010	0.26	0.633	1.75	0.485	12.10	0.002	0.36	0.693
C3	2.81	0.993	22.44	0.000	4.79	0.169	6.17	0.356	46.70	0.000	13.67	0.008
C4	0.86	0.993	15.13	0.001	1.13	0.364	4.22	0.356	19.10	0.000	2.03	0.261
C5	1.02	0.993	13.29	0.001	2.78	0.215	0.52	0.625	9.29	0.007	2.56	0.234
C6	0.61	0.993	18.83	0.000	1.92	0.277	0.73	0.587	22.39	0.000	3.49	0.168
T7	0.32	0.993	10.67	0.003	0.95	0.397	0.01	0.925	4.47	0.049	0.08	0.834
T8	0.12	0.993	13.69	0.001	0.33	0.602	0.07	0.844	2.91	0.109	0.01	0.906
CP1	0.00	0.993	1.27	0.282	0.39	0.578	0.78	0.587	1.80	0.199	0.19	0.782
CP2	0.00	0.993	0.82	0.378	0.60	0.493	1.67	0.485	1.64	0.217	0.17	0.782
CP3	2.53	0.993	16.30	0.000	3.45	0.192	5.54	0.356	23.96	0.000	6.85	0.057
CP4	0.74	0.993	18.41	0.000	1.24	0.348	3.84	0.386	15.04	0.001	2.16	0.261
CP5	0.64	0.993	23.89	0.000	4.27	0.173	0.88	0.587	18.56	0.000	6.92	0.057

CP6	1.86	0.993	26.78	0.000	4.52	0.171	2.29	0.485	21.70	0.000	5.20	0.109
TP7	0.49	0.993	16.60	0.000	0.61	0.493	0.05	0.870	4.99	0.039	0.04	0.882
TP8	0.81	0.993	20.72	0.000	1.92	0.277	0.77	0.587	4.48	0.049	0.38	0.693
Pz	0.31	0.993	1.23	0.285	0.00	0.973	0.34	0.674	0.61	0.440	0.02	0.891
P3	0.41	0.993	7.29	0.012	2.23	0.252	1.67	0.485	5.17	0.037	4.54	0.134
P4	0.67	0.993	11.82	0.002	1.89	0.277	3.44	0.418	7.40	0.014	3.39	0.170
P5	0.06	0.993	18.54	0.000	2.65	0.216	0.08	0.844	14.72	0.001	2.44	0.236
P6	0.37	0.993	27.31	0.000	3.38	0.192	1.87	0.485	11.43	0.003	4.29	0.137
P7	0.24	0.993	28.22	0.000	3.27	0.192	0.40	0.674	17.89	0.000	2.11	0.261
P8	0.07	0.993	30.25	0.000	3.69	0.192	0.30	0.674	16.78	0.001	1.45	0.333
POz	0.13	0.993	12.21	0.002	2.50	0.221	2.59	0.463	2.02	0.181	2.04	0.261
PO3	0.00	0.993	16.80	0.000	1.75	0.288	1.25	0.535	5.44	0.033	1.04	0.422
PO4	0.04	0.993	14.43	0.001	1.82	0.282	1.96	0.485	1.96	0.184	3.07	0.196
PO5	0.05	0.993	24.89	0.000	0.50	0.528	0.28	0.674	13.05	0.002	0.16	0.782
PO6	0.04	0.993	18.20	0.000	2.11	0.264	1.47	0.507	4.07	0.059	2.91	0.205
PO7	0.00	0.993	26.54	0.000	1.53	0.309	0.01	0.925	12.38	0.002	0.14	0.782
PO8	0.09	0.993	40.30	0.000	3.73	0.192	1.33	0.525	11.77	0.003	1.99	0.262
Oz	0.50	0.993	38.61	0.000	7.96	0.096	2.96	0.463	7.65	0.013	2.23	0.259
O1	0.01	0.993	29.45	0.000	3.79	0.192	0.80	0.587	8.85	0.008	0.06	0.857
O2	0.13	0.993	32.55	0.000	6.00	0.131	1.04	0.580	5.43	0.033	1.85	0.277

Table S4.The results of two-factor analysis of variance

Channels	delta						theta					
	group		session		Interaction effect		group		session		Interaction effect	
	F	P'	F	P'	F	P'	F	P'	F	P'	F	P'
Fpz	0.00	0.991	15.27	0.001	1.25	0.347	0.05	0.992	24.47	0.000	3.31	0.110
Fp1	0.01	0.983	21.69	0.000	2.30	0.234	0.03	0.992	37.59	0.000	4.16	0.084
Fp2	0.54	0.710	13.54	0.002	3.09	0.178	0.34	0.949	23.84	0.000	7.53	0.034
AF3	0.12	0.844	22.30	0.000	5.08	0.088	0.00	0.992	48.20	0.000	10.47	0.014
AF4	0.14	0.844	31.06	0.000	8.18	0.048	0.01	0.992	45.66	0.000	11.78	0.014
AF7	1.06	0.627	12.49	0.003	1.22	0.347	0.89	0.811	34.17	0.000	4.41	0.076
AF8	1.25	0.627	12.90	0.002	1.36	0.330	1.83	0.607	28.74	0.000	5.97	0.054
Fz	1.37	0.627	20.42	0.000	4.03	0.131	0.02	0.992	24.70	0.000	3.39	0.108
F1	2.65	0.415	31.97	0.000	19.68	0.003	0.45	0.905	43.17	0.000	11.01	0.014
F2	0.89	0.627	29.94	0.000	9.24	0.040	0.00	0.992	31.07	0.000	6.65	0.043
F3	2.90	0.386	48.13	0.000	17.50	0.004	1.63	0.624	81.26	0.000	17.87	0.004
F4	1.75	0.566	26.51	0.000	7.27	0.055	0.64	0.816	40.51	0.000	10.47	0.014
F5	0.20	0.805	20.44	0.000	6.39	0.062	0.49	0.901	44.27	0.000	9.91	0.015
F6	1.28	0.627	10.87	0.004	0.38	0.602	2.19	0.553	28.66	0.000	3.00	0.125
F7	0.29	0.805	5.52	0.034	2.99	0.178	0.68	0.816	21.14	0.000	9.87	0.015
F8	1.13	0.627	5.41	0.035	0.02	0.887	3.49	0.380	21.85	0.000	1.32	0.299

FCz	0.92	0.627	7.71	0.013	2.54	0.216	0.00	0.992	7.04	0.015	0.00	0.965
FC1	4.64	0.224	14.58	0.002	8.00	0.048	1.73	0.615	23.59	0.000	2.51	0.154
FC2	3.99	0.246	5.04	0.041	1.50	0.315	0.82	0.811	11.63	0.002	0.81	0.410
FC3	13.69	0.026	35.59	0.000	24.49	0.001	8.60	0.091	62.98	0.000	22.97	0.001
FC4	4.94	0.221	13.92	0.002	4.75	0.099	3.90	0.336	23.49	0.000	3.76	0.095
FC5	2.29	0.435	5.40	0.035	4.46	0.109	2.23	0.553	11.45	0.002	5.34	0.058
FC6	3.60	0.282	8.75	0.009	1.44	0.321	5.07	0.231	20.12	0.000	3.47	0.106
FT7	2.34	0.435	0.95	0.376	2.98	0.178	3.19	0.411	5.93	0.024	6.73	0.043
FT8	9.09	0.049	4.92	0.042	2.33	0.234	12.22	0.046	21.51	0.000	4.55	0.073
Cz	0.12	0.844	0.62	0.479	1.59	0.315	0.89	0.811	0.26	0.628	4.90	0.063
C1	1.11	0.627	0.16	0.706	2.60	0.215	0.04	0.992	0.63	0.465	0.00	0.988
C2	1.23	0.627	2.56	0.138	1.74	0.314	0.27	0.953	5.53	0.028	1.86	0.224
C3	13.42	0.026	9.51	0.007	14.60	0.009	8.99	0.091	25.82	0.000	14.02	0.008
C4	11.78	0.033	16.95	0.001	7.45	0.055	6.20	0.179	23.73	0.000	5.79	0.056
C5	4.92	0.221	4.87	0.042	5.70	0.075	2.18	0.553	7.79	0.011	4.03	0.087
C6	4.32	0.226	14.23	0.002	6.90	0.060	2.55	0.545	20.88	0.000	4.95	0.063
T7	0.74	0.652	0.27	0.636	0.23	0.683	0.17	0.992	1.75	0.217	0.36	0.574
T8	1.01	0.627	2.85	0.120	1.53	0.315	0.27	0.953	7.84	0.011	0.44	0.550
CP1	0.03	0.947	0.02	0.903	0.09	0.782	0.00	0.992	0.04	0.853	0.89	0.391
CP2	0.86	0.627	0.37	0.588	0.49	0.566	0.10	0.992	0.45	0.527	1.06	0.354
CP3	9.43	0.049	7.79	0.013	9.95	0.039	7.63	0.111	18.69	0.000	12.92	0.011
CP4	7.61	0.080	21.43	0.000	6.25	0.062	4.15	0.328	34.11	0.000	6.28	0.049
CP5	4.50	0.224	12.03	0.003	9.62	0.039	2.02	0.571	26.26	0.000	15.10	0.007
CP6	10.59	0.040	7.98	0.013	7.95	0.048	5.36	0.230	26.34	0.000	9.71	0.015
TP7	1.70	0.566	1.88	0.204	0.10	0.779	0.74	0.816	8.88	0.007	1.52	0.273
TP8	2.56	0.415	6.30	0.024	5.18	0.088	0.99	0.811	13.48	0.001	2.91	0.128
Pz	0.73	0.652	0.18	0.702	0.14	0.746	0.85	0.811	0.49	0.515	0.41	0.554
P3	0.68	0.663	3.73	0.075	6.35	0.062	0.71	0.816	11.56	0.002	8.92	0.020
P4	0.59	0.698	7.87	0.013	1.51	0.315	0.84	0.811	24.77	0.000	4.89	0.063
P5	0.21	0.805	16.93	0.001	6.29	0.062	0.24	0.953	35.15	0.000	11.41	0.014
P6	0.97	0.627	7.75	0.013	3.40	0.169	1.13	0.811	46.30	0.000	10.47	0.014
P7	1.15	0.627	13.28	0.002	1.69	0.314	0.39	0.930	34.38	0.000	5.64	0.056
P8	0.09	0.864	11.13	0.004	5.34	0.085	0.12	0.992	30.50	0.000	6.99	0.041
POz	0.45	0.750	12.30	0.003	0.45	0.575	0.24	0.953	22.34	0.000	1.49	0.274
PO3	0.00	0.991	11.69	0.003	0.75	0.472	0.01	0.992	24.00	0.000	3.62	0.100
PO4	0.20	0.805	13.29	0.002	1.59	0.315	0.04	0.992	40.33	0.000	3.94	0.089
PO5	0.22	0.805	14.31	0.002	0.33	0.620	0.05	0.992	33.67	0.000	3.11	0.120
PO6	0.20	0.805	19.66	0.000	3.26	0.175	0.00	0.992	40.87	0.000	5.33	0.058
PO7	0.00	0.991	17.43	0.001	0.58	0.533	0.00	0.992	32.41	0.000	2.82	0.132
PO8	0.04	0.936	24.03	0.000	1.76	0.314	0.02	0.992	53.04	0.000	4.93	0.063

Oz	0.01	0.983	24.58	0.000	3.06	0.178	0.02	0.992	60.93	0.000	5.51	0.057
O1	0.21	0.805	25.81	0.000	1.08	0.378	0.06	0.992	59.16	0.000	5.71	0.056
O2	0.23	0.805	23.48	0.000	3.47	0.169	11.94	0.046	0.80	0.413	21.88	0.001

Table S5 Post hoc test for the average power of the differential channel (Group)

Channels	delta		theta	
	t	p	t	p
FC3	3.40	0.001		
FT8	3.25	0.002	3.63	0.001
C3	3.75	0.000		
C4	3.53	0.001		
CP3	3.28	0.002		
CP6	3.19	0.002		
O2			-4.16	0.000

Table S6 Post hoc test for the average power of the differential channel (session)

Channels	delta		theta		alpha		beta	
	t	p	t	p	t	p	t	p
Fpz	3.02	0.004	3.39	0.001	3.39	0.001	3.88	0.000
Fp1	3.21	0.002	3.97	0.000	3.82	0.000	3.86	0.000
Fp2	2.61	0.011	2.92	0.005	2.90	0.005	3.05	0.003
AF3	3.72	0.000	4.16	0.000	4.00	0.000	4.23	0.000
AF4	3.50	0.001	3.46	0.001	3.50	0.001	4.21	0.000
AF7	2.85	0.006	4.22	0.000	4.19	0.000	3.64	0.001
AF8	2.76	0.008	3.23	0.002	3.33	0.001	3.44	0.001
Fz	3.15	0.002	2.93	0.005	3.45	0.001	4.43	0.000
F1	3.86	0.000	3.54	0.001	3.76	0.000	4.59	0.000
F2	3.42	0.001	3.01	0.004	3.52	0.001	4.41	0.000
F3	4.65	0.000	4.90	0.000	4.82	0.000	5.22	0.000
F4	3.63	0.001	3.63	0.001	4.02	0.000	4.24	0.000
F5	3.23	0.002	4.50	0.000	4.48	0.000	3.88	0.000
F6	2.56	0.013	3.46	0.001	3.52	0.001	3.69	0.000
F7	1.76	0.082	3.18	0.002	3.58	0.001	3.12	0.003
F8	2.06	0.044	3.52	0.001	3.55	0.001	3.46	0.001
FCz	1.67	0.100	1.46	0.150	2.66	0.010	3.33	0.001
FC1	2.73	0.008	2.68	0.009	3.03	0.004	3.73	0.000
FC2	1.69	0.096	2.13	0.037	3.02	0.004	3.26	0.002
FC3	3.99	0.000	4.51	0.000	3.81	0.000	4.68	0.000
FC4	2.94	0.005	3.38	0.001	3.58	0.001	4.30	0.000
FC5	1.87	0.066	2.64	0.010	2.68	0.009	2.29	0.025
FC6	2.43	0.018	3.32	0.001	3.40	0.001	3.83	0.000
FT7	0.00	1.000	1.80	0.076	2.72	0.008	2.41	0.019

FT8	1.81	0.075	3.38	0.001	3.75	0.000	3.16	0.002
Cz	0.00	1.000	0.00	1.000	0.00	1.000	0.00	1.000
C1	0.00	1.000	0.00	1.000	0.00	1.000	2.07	0.042
C2	0.00	1.000	1.38	0.172	1.62	0.110	2.03	0.046
C3	2.21	0.031	3.02	0.004	2.58	0.012	3.22	0.002
C4	3.04	0.003	3.07	0.003	2.31	0.024	3.05	0.003
C5	1.90	0.062	2.22	0.030	2.37	0.021	2.21	0.031
C6	2.63	0.011	3.03	0.004	2.65	0.010	2.94	0.005
T7	0.00	1.000	0.00	1.000	1.83	0.072	1.37	0.176
T8	0.00	1.000	2.01	0.048	2.20	0.031	0.00	1.000
CP1	0.00	1.000	0.00	1.000	0.00	1.000	0.00	1.000
CP2	0.00	1.000	0.00	1.000	0.00	1.000	0.00	1.000
CP3	2.06	0.044	2.76	0.008	2.42	0.018	2.48	0.016
CP4	3.40	0.001	3.36	0.001	2.60	0.011	2.79	0.007
CP5	2.48	0.016	2.97	0.004	2.88	0.005	2.60	0.011
CP6	2.35	0.022	3.41	0.001	3.29	0.002	2.83	0.006
TP7	0.00	1.000	1.72	0.091	2.24	0.028	1.62	0.111
TP8	1.86	0.068	2.73	0.008	3.14	0.003	1.64	0.106
Pz	0.00	1.000	0.00	1.000	0.00	1.000	0.00	1.000
P3	0.00	1.000	2.04	0.045	1.72	0.089	1.27	0.209
P4	1.88	0.064	2.47	0.016	1.96	0.054	1.74	0.087
P5	2.94	0.005	3.42	0.001	2.53	0.014	2.08	0.042
P6	1.87	0.066	3.33	0.001	2.55	0.013	2.04	0.046
P7	3.00	0.004	3.50	0.001	3.19	0.002	3.08	0.003
P8	2.39	0.020	3.41	0.001	3.16	0.002	2.77	0.007
POz	2.03	0.046	2.28	0.026	1.99	0.051	0.00	1.000
PO3	2.33	0.023	2.81	0.006	2.35	0.022	1.26	0.212
PO4	2.22	0.030	2.85	0.006	2.22	0.030	0.00	1.000
PO5	3.23	0.002	3.89	0.000	2.95	0.004	2.15	0.035
PO6	2.83	0.006	3.53	0.001	2.65	0.010	0.00	1.000
PO7	3.54	0.001	3.84	0.000	3.24	0.002	2.43	0.018
PO8	3.74	0.000	4.22	0.000	3.76	0.000	2.46	0.017
Oz	3.61	0.001	4.41	0.000	3.30	0.002	1.81	0.076
O1	3.82	0.000	4.58	0.000	3.46	0.001	2.32	0.023
O2	3.53	0.001	0.00	1.000	3.52	0.001	1.89	0.063

Table S7 Post hoc test for channels with interaction effects

Channels	tDCS:sd v rs		BC:sd v rs		SD:tDCS v BC		RS:tDCS v BC	
	t	p	t	p	t	p	t	p
delta								
AF4	7.21	0.000	1.67	0.114	1.36	0.182	-1.24	0.223
F1	6.22	0.000	1.04	0.314	3.18	0.003	-1.21	0.235
F2	5.10	0.000	2.21	0.042	1.98	0.056	-0.62	0.541
F3	8.19	0.000	1.88	0.079	3.37	0.002	-0.74	0.464

FC1	3.75	0.002	1.07	0.300	3.13	0.004	0.31	0.762
FC3	6.39	0.000	0.98	0.342	5.47	0.000	0.17	0.864
C3	3.94	0.001	-0.76	0.457	5.33	0.000	0.65	0.520
CP3	3.51	0.003	-0.34	0.737	4.00	0.000	0.71	0.484
CP5	3.77	0.002	0.37	0.714	3.01	0.005	0.11	0.909
CP6	3.15	0.006	0.01	0.995	3.73	0.001	0.72	0.477
				theta				
Fp2	5.08	0.000	1.62	0.125	1.49	0.146	-0.77	0.446
AF3	7.56	0.000	2.51	0.023	1.15	0.258	-1.81	0.080
AF4	6.36	0.000	2.78	0.014	1.14	0.262	-1.49	0.146
F1	6.18	0.000	2.71	0.016	1.64	0.112	-0.83	0.415
F2	4.64	0.000	3.14	0.006	0.88	0.387	-1.13	0.266
F3	9.64	0.000	3.29	0.005	2.71	0.011	-0.57	0.572
F4	5.49	0.000	3.23	0.005	1.83	0.076	-0.70	0.492
F5	7.75	0.000	2.26	0.038	1.88	0.069	-1.13	0.265
F7	5.07	0.000	1.13	0.277	2.26	0.031	-0.89	0.381
FC3	7.48	0.000	2.99	0.009	4.48	0.000	0.42	0.679
FT7	3.12	0.007	-0.13	0.894	2.58	0.015	0.08	0.937
C3	5.09	0.000	1.34	0.199	4.31	0.000	0.88	0.384
CP3	4.44	0.000	0.80	0.434	3.63	0.001	0.72	0.479
CP4	4.50	0.000	4.44	0.000	2.56	0.015	0.92	0.367
CP5	4.87	0.000	1.62	0.125	2.46	0.019	-0.52	0.606
CP6	4.72	0.000	2.07	0.055	3.02	0.005	0.53	0.599
P3	3.56	0.003	0.47	0.645	1.77	0.086	-0.65	0.517
P5	5.40	0.000	2.52	0.023	1.60	0.120	-1.18	0.247
P6	5.97	0.000	3.30	0.005	2.10	0.044	-0.15	0.881
P8	5.22	0.000	2.31	0.035	1.32	0.197	-0.99	0.332
O2	-2.97	0.009	5.48	0.000	-4.41	0.000	-1.27	0.212
				beta				
Fp1	6.13	0.000	1.71	0.106	1.91	0.066	-0.91	0.371
AF3	9.93	0.000	2.76	0.014	1.96	0.058	-0.78	0.443
AF4	6.02	0.000	3.32	0.004	1.57	0.127	-0.89	0.379
Fz	7.53	0.000	3.41	0.004	2.23	0.033	-0.30	0.769
F1	7.30	0.000	3.44	0.003	2.76	0.010	-0.50	0.622
F2	7.41	0.000	3.26	0.005	2.26	0.031	-0.95	0.351
F3	8.26	0.000	3.23	0.005	2.91	0.007	-0.54	0.590
F4	5.99	0.000	3.51	0.003	1.99	0.055	-0.75	0.459
F5	6.69	0.000	1.90	0.076	1.61	0.118	-1.33	0.192
F7	5.27	0.000	1.23	0.237	1.83	0.077	-0.91	0.368
FC3	7.91	0.000	3.10	0.007	4.17	0.000	0.68	0.500
C3	6.01	0.000	3.25	0.005	3.56	0.001	1.00	0.326

Table S8 A power mean table of channels with interactive effects

Channels	tDCS SD		tDCS RS		BC SD		BC RS	
	mean	sd	mean	sd	mean	sd	mean	sd
delta								
AF4	6.28	2.57	3.22	1.40	4.97	3.03	3.99	2.12
F1	5.73	2.09	2.43	1.40	3.42	2.15	3.02	1.46
F2	5.49	2.32	2.70	1.99	3.89	2.38	3.09	1.65
F3	5.90	2.32	1.59	1.88	3.18	2.40	2.11	2.18
FC1	3.92	2.18	1.42	2.15	1.59	2.14	1.22	1.56
FC3	4.97	2.56	0.44	2.10	0.75	1.89	0.33	1.72
C3	3.11	2.18	-0.11	2.18	-0.97	2.28	-0.63	2.47
CP3	3.28	2.58	0.80	1.79	0.20	1.86	0.35	1.97
CP5	3.64	3.25	0.75	2.16	0.84	2.04	0.68	1.67
CP6	3.80	2.84	1.03	2.06	0.60	2.10	0.60	1.43
theta								
Fp2	-0.03	3.01	-3.56	1.99	-1.87	4.11	-2.86	3.16
AF3	-0.89	2.36	-4.47	1.63	-2.00	3.20	-3.30	2.11
AF4	-0.73	2.53	-3.89	1.23	-1.87	3.27	-2.90	2.44
F1	-1.01	2.10	-3.88	1.65	-2.44	2.93	-3.38	1.86
F2	-1.28	2.32	-3.74	1.92	-2.05	2.77	-2.95	2.10
F3	-0.88	2.35	-5.10	1.90	-3.15	2.53	-4.67	2.39
F4	-1.10	2.20	-4.14	1.42	-2.69	2.81	-3.68	2.34
F5	-1.46	2.84	-5.23	1.50	-3.15	2.37	-4.50	2.20
F7	-0.67	3.02	-4.12	2.69	-2.70	2.12	-3.35	2.36
FC3	-1.78	2.43	-6.24	1.73	-5.42	2.32	-6.52	2.28
FT7	-1.59	3.97	-4.27	2.49	-4.42	2.17	-4.34	2.30
C3	-3.57	2.28	-6.78	1.94	-6.95	2.30	-7.44	2.39
CP3	-3.12	2.82	-6.09	1.58	-6.25	2.18	-6.53	1.95
CP4	-3.64	2.77	-6.43	1.63	-5.91	2.39	-7.03	2.15
CP5	-2.68	3.49	-5.94	1.96	-5.16	2.25	-5.61	1.68
CP6	-2.65	2.55	-5.72	1.44	-5.27	2.51	-6.02	1.85
P3	-2.25	2.97	-4.39	1.81	-3.84	2.21	-3.98	1.85
P5	-1.60	2.61	-4.40	1.74	-2.94	2.25	-3.71	1.70
P6	-1.18	1.94	-3.58	2.27	-2.63	2.07	-3.48	1.52
P8	-0.90	2.22	-3.41	1.65	-1.93	2.32	-2.81	1.88
O2	-6.22	3.47	-4.03	2.00	-1.66	2.47	-3.15	2.05
beta								
Fp1	-6.13	2.52	-9.93	1.95	-8.06	3.31	-9.24	2.42
AF3	-7.81	2.14	-11.04	1.45	-9.35	2.41	-10.53	2.28
AF4	-7.64	2.15	-10.78	1.57	-8.94	2.67	-10.22	2.02
Fz	-8.60	1.82	-11.65	1.60	-10.24	2.41	-11.48	1.82
F1	-8.11	2.40	-11.83	1.53	-10.36	2.37	-11.54	1.87
F2	-8.48	1.89	-11.75	1.75	-10.12	2.32	-11.18	1.80

F3	-7.62	2.08	-11.91	1.85	-9.90	2.46	-11.51	2.39
F4	-7.79	2.06	-11.09	1.85	-9.35	2.48	-10.57	2.17
F5	-8.48	2.33	-11.46	1.58	-9.64	1.82	-10.56	2.33
F7	-7.61	2.55	-10.55	2.12	-9.13	2.26	-9.82	2.48
FC3	-8.92	1.99	-12.85	1.93	-11.99	2.30	-13.31	1.99
C3	-10.24	2.51	-13.31	2.16	-13.17	2.27	-14.08	2.37

Table S9 Results of EEG correlation of facial expression in electrical stimulation group

Wave	Channels	neutral		Happy		sad		angry	
		rho	p	rho	p	rho	p	rho	p
alpha	F1	0.01	0.981	-0.56	0.020	0.33	0.191	0.15	0.559
	F3	0.03	0.906	-0.23	0.377	0.26	0.308	0.18	0.485
	F4	-0.17	0.509	-0.43	0.083	0.46	0.066	0.07	0.802
	F5	0.02	0.943	-0.29	0.251	-0.09	0.730	0.27	0.294
	F7	0.01	0.981	0.00	0.996	-0.21	0.426	0.29	0.259
	FC3	0.19	0.461	-0.02	0.928	0.06	0.809	-0.02	0.951
	FT7	0.05	0.854	-0.08	0.758	-0.18	0.485	0.34	0.178
	OZ	0.04	0.891	0.04	0.883	0.11	0.687	-0.04	0.883
	O2	-0.08	0.751	0.08	0.766	0.25	0.332	0.00	0.996
beta	FP1	-0.03	0.898	-0.36	0.153	-0.01	0.974	-0.01	0.974
	AF3	-0.21	0.415	-0.67	0.004	0.33	0.201	0.01	0.959
	AF4	-0.05	0.839	-0.69	0.003	0.21	0.421	0.09	0.723
	FZ	0.25	0.322	-0.13	0.625	-0.04	0.869	0.36	0.159
	F1	0.24	0.352	0.18	0.497	-0.19	0.461	0.31	0.231
	F2	0.28	0.276	0.03	0.906	-0.03	0.913	0.19	0.461
	F3	-0.01	0.974	-0.06	0.817	0.19	0.455	0.17	0.521
	F4	-0.16	0.540	-0.29	0.255	0.27	0.299	0.18	0.497
	F5	0.11	0.673	-0.34	0.188	-0.15	0.559	0.30	0.239
	F7	0.06	0.817	0.25	0.332	-0.23	0.377	0.04	0.876
	FC2	0.17	0.521	-0.06	0.831	-0.13	0.605	0.35	0.171
	FC3	0.48	0.052	0.16	0.547	-0.22	0.404	0.04	0.883
	C3	0.15	0.572	0.06	0.817	0.16	0.534	0.12	0.646
	CP3	0.37	0.147	0.14	0.579	-0.09	0.744	-0.05	0.854
	CP5	-0.02	0.928	-0.05	0.854	-0.17	0.515	-0.01	0.981
delta	AF4	-0.15	0.553	-0.56	0.022	0.21	0.415	-0.12	0.646
	F1	0.02	0.951	-0.18	0.497	0.30	0.235	-0.04	0.876
	F2	0.06	0.831	-0.12	0.653	0.06	0.809	-0.10	0.715
	F3	-0.07	0.787	-0.18	0.485	0.32	0.205	-0.02	0.936
	F4	0.01	0.959	-0.07	0.795	0.11	0.680	0.07	0.795
	F5	0.18	0.479	-0.13	0.619	-0.22	0.399	0.07	0.802
	FC1	0.41	0.102	0.04	0.869	0.04	0.891	-0.34	0.181
	FC3	0.33	0.198	0.21	0.415	-0.06	0.831	-0.18	0.497
	C3	0.37	0.150	0.03	0.906	0.05	0.846	0.01	0.981
	C4	-0.06	0.824	0.19	0.455	0.15	0.572	-0.10	0.701
	C5	0.25	0.337	0.04	0.891	-0.26	0.303	-0.01	0.981
	C6	0.25	0.337	-0.04	0.883	-0.23	0.377	0.06	0.831
	CP3	0.38	0.136	0.07	0.780	-0.03	0.921	-0.14	0.592
	CP4	0.16	0.534	0.39	0.120	-0.13	0.625	-0.22	0.388
	CP5	0.03	0.921	-0.01	0.966	-0.13	0.632	-0.08	0.766
	CP6	0.40	0.109	0.18	0.485	-0.37	0.150	-0.31	0.223
	P3	0.10	0.708	0.31	0.227	0.11	0.673	-0.31	0.231

	P5	0.21	0.421	-0.15	0.553	0.10	0.701	-0.34	0.184
	FP2	-0.33	0.198	-0.66	0.005	0.34	0.181	-0.04	0.876
	AF3	-0.24	0.357	-0.65	0.006	0.31	0.231	0.12	0.653
	AF4	-0.07	0.802	-0.74	0.001	0.25	0.327	-0.12	0.653
	AF8	0.17	0.521	-0.52	0.033	0.15	0.553	-0.24	0.357
	F1	-0.23	0.383	-0.54	0.026	0.39	0.118	0.20	0.432
	F2	0.00	0.996	-0.40	0.111	0.10	0.708	0.16	0.547
	F3	-0.15	0.559	-0.18	0.497	0.29	0.259	0.19	0.455
	F4	-0.17	0.515	-0.34	0.188	0.21	0.410	0.10	0.694
	F5	-0.11	0.680	-0.29	0.264	-0.06	0.831	0.21	0.421
	F7	-0.10	0.715	0.20	0.438	-0.29	0.264	0.19	0.461
	FC3	0.23	0.367	-0.08	0.751	0.04	0.883	0.09	0.744
theta	FT7	-0.09	0.744	0.10	0.701	-0.33	0.191	0.27	0.290
	C3	0.23	0.377	-0.29	0.255	0.29	0.259	0.12	0.639
	C4	-0.36	0.162	-0.06	0.831	0.39	0.118	-0.05	0.846
	CP3	0.33	0.191	-0.02	0.928	0.01	0.974	0.04	0.883
	CP4	-0.01	0.959	0.17	0.509	0.10	0.701	-0.07	0.795
	CP5	0.07	0.780	-0.21	0.426	-0.06	0.817	-0.05	0.861
	CP6	0.23	0.383	0.00	0.996	-0.06	0.824	-0.26	0.308
	P3	-0.05	0.861	-0.03	0.913	0.15	0.566	-0.02	0.936
	P5	0.02	0.943	-0.43	0.087	0.27	0.294	-0.28	0.276
	P6	-0.40	0.109	0.05	0.854	0.33	0.191	0.00	0.996
	P8	-0.15	0.559	-0.63	0.009	0.34	0.178	0.15	0.566
	O1	-0.02	0.928	-0.08	0.766	0.01	0.974	-0.08	0.758
	O2	0.05	0.861	-0.06	0.824	-0.07	0.787	0.08	0.773

Table S10 Results of EEG correlation of facial expression in electrical stimulation group

Wave	Channels	surprised		scared		disgusted	
		rho	p	rho	p	rho	p
	F1	-0.40	0.109	0.14	0.592	-0.11	0.687
	F3	-0.29	0.251	0.11	0.687	0.20	0.443
	F4	-0.24	0.362	0.46	0.066	-0.14	0.592
	F5	-0.17	0.509	0.25	0.327	-0.08	0.773
alpha	F7	0.07	0.787	0.53	0.032	-0.03	0.898
	FC3	-0.23	0.377	0.08	0.751	-0.25	0.337
	FT7	-0.04	0.891	0.50	0.045	-0.08	0.773
	OZ	-0.20	0.432	0.13	0.625	-0.09	0.730
	O2	-0.46	0.068	0.42	0.095	0.06	0.824
	FP1	-0.09	0.744	-0.21	0.415	-0.04	0.883
beta	AF3	-0.40	0.109	0.00	0.989	-0.32	0.212
	AF4	-0.32	0.212	0.18	0.479	0.00	0.996
	FZ	-0.07	0.787	-0.03	0.921	0.16	0.528

	F1	-0.09	0.737	0.03	0.921	0.28	0.276
	F2	0.02	0.943	0.12	0.653	0.08	0.773
	F3	-0.32	0.216	0.07	0.802	0.30	0.243
	F4	-0.26	0.303	0.33	0.198	0.00	0.996
	F5	-0.05	0.861	0.03	0.906	0.07	0.795
	F7	0.08	0.773	0.43	0.083	0.12	0.653
	FC2	-0.19	0.467	-0.01	0.966	-0.09	0.744
	FC3	-0.11	0.687	-0.12	0.653	-0.05	0.839
	C3	-0.20	0.443	-0.04	0.891	0.11	0.673
	CP3	-0.02	0.936	-0.18	0.491	-0.33	0.201
	CP5	0.31	0.223	0.23	0.377	-0.33	0.201
	AF4	-0.01	0.959	0.07	0.802	-0.32	0.216
	F1	0.14	0.579	0.07	0.795	-0.11	0.687
	F2	0.19	0.473	0.05	0.846	-0.15	0.572
	F3	-0.10	0.701	0.03	0.913	0.02	0.943
	F4	0.12	0.639	0.26	0.317	-0.16	0.528
	F5	0.00	0.996	0.21	0.426	-0.12	0.646
	FC1	0.06	0.824	-0.20	0.438	-0.55	0.023
	FC3	0.00	0.996	-0.05	0.839	-0.35	0.171
delta	C3	0.09	0.737	-0.25	0.327	-0.26	0.313
	C4	-0.03	0.906	0.13	0.619	-0.10	0.701
	C5	0.12	0.639	0.22	0.404	-0.35	0.168
	C6	0.09	0.744	0.20	0.438	-0.28	0.268
	CP3	0.13	0.619	-0.18	0.491	-0.50	0.045
	CP4	0.05	0.861	0.07	0.787	-0.11	0.687
	CP5	0.47	0.057	0.12	0.639	-0.53	0.029
	CP6	0.13	0.632	-0.07	0.780	-0.32	0.209
	P3	0.16	0.528	0.19	0.473	-0.40	0.116
	P5	0.12	0.653	0.21	0.426	-0.58	0.016
	FP2	-0.17	0.503	0.11	0.673	-0.15	0.572
	AF3	-0.07	0.795	0.04	0.891	-0.37	0.141
	AF4	-0.07	0.787	0.03	0.906	-0.42	0.095
	AF8	-0.19	0.467	0.20	0.449	-0.22	0.404
	F1	-0.05	0.846	0.05	0.846	-0.19	0.461
	F2	0.15	0.553	0.07	0.787	-0.15	0.559
	F3	-0.21	0.426	0.10	0.701	0.06	0.831
theta	F4	0.18	0.497	0.20	0.449	-0.25	0.322
	F5	-0.10	0.694	0.10	0.715	-0.07	0.802
	F7	0.29	0.255	0.48	0.056	-0.06	0.831
	FC3	-0.11	0.666	-0.18	0.497	-0.25	0.327
	FT7	0.11	0.673	0.45	0.074	-0.01	0.966
	C3	-0.15	0.572	-0.22	0.404	-0.19	0.461
	C4	-0.37	0.141	0.29	0.264	-0.03	0.898
	CP3	-0.06	0.824	-0.12	0.646	-0.31	0.227

CP4	-0.13	0.625	0.23	0.383	-0.12	0.639
CP5	0.24	0.362	0.20	0.432	-0.56	0.022
CP6	-0.28	0.276	0.03	0.906	-0.23	0.367
P3	0.04	0.876	0.04	0.891	-0.27	0.294
P5	-0.02	0.943	0.25	0.337	-0.64	0.006
P6	-0.15	0.566	0.53	0.031	-0.13	0.619
P8	-0.30	0.235	0.48	0.056	-0.21	0.421
O1	0.14	0.592	0.04	0.876	-0.39	0.126
O2	-0.21	0.415	-0.36	0.156	0.05	0.839

Table S11 Results of EEg correlation of facial expression in blank group

Wave	Channels	neutral		Happy		sad		angry	
		rho	p	rho	p	rho	p	rho	p
alpha	F1	0.09	0.737	-0.28	0.276	-0.19	0.473	0.08	0.758
	F3	-0.15	0.572	-0.03	0.898	-0.27	0.285	0.16	0.528
	F4	-0.22	0.404	-0.36	0.153	-0.24	0.352	0.28	0.276
	F5	-0.16	0.528	-0.25	0.337	0.01	0.974	0.01	0.981
	F7	0.03	0.906	-0.19	0.455	-0.22	0.399	0.08	0.773
	FC3	0.05	0.861	-0.10	0.715	0.03	0.898	0.07	0.795
	FT7	-0.29	0.264	-0.31	0.227	0.17	0.509	-0.09	0.744
	OZ	0.00	0.996	-0.25	0.342	-0.13	0.632	-0.02	0.943
	O2	0.09	0.730	-0.34	0.188	0.07	0.780	-0.12	0.660
beta	FP1	0.18	0.479	-0.13	0.625	-0.20	0.449	-0.31	0.231
	AF3	0.21	0.421	0.09	0.744	-0.35	0.171	-0.17	0.509
	AF4	0.33	0.198	-0.26	0.308	-0.16	0.528	-0.20	0.449
	FZ	0.14	0.579	-0.31	0.231	-0.37	0.147	0.19	0.455
	F1	0.35	0.174	-0.19	0.455	-0.16	0.534	-0.06	0.831
	F2	0.10	0.701	-0.30	0.243	-0.30	0.239	0.21	0.421
	F3	-0.02	0.936	0.00	0.989	-0.38	0.139	0.09	0.737
	F4	-0.29	0.259	-0.13	0.632	-0.16	0.547	0.10	0.708
	F5	-0.13	0.625	0.03	0.906	-0.13	0.632	-0.18	0.485
	F7	-0.04	0.876	-0.08	0.773	-0.18	0.485	0.18	0.497
	FC2	0.21	0.410	-0.39	0.123	-0.20	0.432	0.26	0.317
	FC3	0.08	0.766	-0.21	0.421	0.04	0.883	0.00	0.989
	C3	-0.04	0.883	-0.23	0.367	0.20	0.438	0.07	0.787
	CP3	0.28	0.268	-0.31	0.227	-0.02	0.951	0.18	0.479
	CP5	0.43	0.085	-0.01	0.966	-0.25	0.327	-0.08	0.751
delta	AF4	-0.11	0.680	-0.54	0.028	0.03	0.913	0.00	0.996
	F1	0.04	0.876	-0.53	0.031	0.07	0.787	0.00	0.989
	F2	0.16	0.540	-0.22	0.388	-0.34	0.188	0.26	0.303
	F3	-0.29	0.259	-0.17	0.503	-0.11	0.687	0.08	0.751
	F4	-0.15	0.553	-0.14	0.585	-0.01	0.981	-0.09	0.730
	F5	-0.36	0.153	-0.35	0.174	0.17	0.503	-0.09	0.730

	FC1	-0.32	0.212	-0.51	0.039	0.10	0.694	0.27	0.285
	FC3	-0.23	0.372	-0.37	0.147	0.26	0.313	-0.03	0.906
	C3	-0.10	0.694	-0.21	0.421	0.67	0.004	0.03	0.913
	C4	-0.34	0.184	0.00	0.989	-0.16	0.547	-0.17	0.509
	C5	-0.32	0.205	-0.42	0.095	0.57	0.019	-0.04	0.869
	C6	-0.22	0.404	-0.03	0.913	-0.03	0.913	-0.40	0.111
	CP3	-0.03	0.898	-0.26	0.317	0.41	0.104	0.14	0.585
	CP4	0.17	0.521	0.17	0.509	-0.51	0.039	-0.10	0.708
	CP5	0.13	0.612	-0.38	0.133	0.29	0.255	0.05	0.854
	CP6	-0.08	0.758	-0.16	0.540	-0.04	0.876	-0.39	0.123
	P3	-0.04	0.876	-0.41	0.104	-0.06	0.809	0.42	0.091
	P5	-0.20	0.443	-0.39	0.118	0.05	0.854	0.43	0.083
	FP2	0.03	0.906	-0.27	0.290	0.09	0.737	-0.28	0.268
theta	AF3	0.05	0.846	0.03	0.898	-0.29	0.251	0.08	0.773
	AF4	0.19	0.461	-0.38	0.139	-0.24	0.362	0.28	0.268
	AF8	0.15	0.559	-0.21	0.426	-0.06	0.831	-0.17	0.521
	F1	0.18	0.491	-0.12	0.646	-0.14	0.599	0.00	0.989
	F2	0.39	0.123	0.07	0.787	-0.52	0.036	0.46	0.066
	F3	-0.30	0.243	-0.12	0.646	-0.12	0.646	0.10	0.694
	F4	-0.13	0.605	-0.11	0.680	-0.51	0.040	0.48	0.053
	F5	-0.28	0.276	-0.32	0.216	0.14	0.585	-0.07	0.780
	F7	-0.14	0.592	-0.18	0.485	-0.12	0.646	0.05	0.839
	FC3	-0.30	0.243	-0.18	0.491	0.28	0.281	-0.11	0.680
	FT7	-0.44	0.076	-0.26	0.317	0.24	0.357	-0.02	0.943
	C3	-0.04	0.891	-0.31	0.223	0.29	0.255	-0.07	0.787
	C4	-0.17	0.509	-0.14	0.579	-0.30	0.247	0.03	0.906
	CP3	0.02	0.936	-0.36	0.162	0.00	0.989	0.21	0.410
	CP4	0.02	0.928	0.32	0.209	-0.60	0.012	-0.04	0.883
	CP5	0.38	0.133	-0.40	0.116	-0.11	0.666	0.12	0.653
	CP6	0.09	0.723	-0.04	0.876	-0.18	0.485	-0.40	0.113
	P3	0.23	0.377	-0.22	0.399	-0.45	0.069	0.14	0.599
	P5	0.04	0.891	-0.27	0.299	-0.31	0.227	0.36	0.162
	P6	0.18	0.479	-0.08	0.773	-0.25	0.327	0.04	0.883
	P8	0.13	0.625	-0.15	0.553	-0.22	0.393	-0.03	0.898
	O1	-0.07	0.795	-0.13	0.625	-0.11	0.666	0.25	0.342
	O2	0.27	0.294	-0.32	0.216	0.06	0.809	0.35	0.165

Table S12 Results of EEg correlation of facial expression in blank group

Wave	Channels	surprised		scared		disgusted	
		rho	p	rho	p	rho	p
alpha	F1	0.15	0.559	0.03	0.921	-0.29	0.259
	F3	0.32	0.205	0.41	0.100	-0.25	0.342

	F4	0.34	0.178	0.29	0.259	0.10	0.715
	F5	0.14	0.585	0.31	0.223	-0.07	0.787
	F7	0.26	0.308	0.26	0.303	0.12	0.646
	FC3	0.14	0.585	0.15	0.572	-0.26	0.308
	FT7	-0.02	0.943	0.03	0.913	0.39	0.120
	OZ	0.21	0.421	0.09	0.723	-0.01	0.974
	O2	0.00	0.996	0.00	0.989	-0.05	0.861
	FP1	0.11	0.687	-0.32	0.212	0.20	0.449
	AF3	0.20	0.449	0.22	0.404	-0.29	0.251
	AF4	0.18	0.497	-0.05	0.846	0.08	0.773
	FZ	0.33	0.194	0.04	0.869	-0.07	0.780
	F1	0.06	0.809	0.05	0.861	-0.33	0.194
	F2	0.25	0.337	0.22	0.393	-0.05	0.839
	F3	0.37	0.141	0.37	0.141	-0.32	0.209
beta	F4	0.08	0.751	0.49	0.049	-0.08	0.751
	F5	0.27	0.294	0.47	0.059	-0.37	0.150
	F7	0.29	0.259	0.40	0.109	0.07	0.795
	FC2	0.18	0.479	0.17	0.509	0.04	0.876
	FC3	-0.02	0.936	0.19	0.461	-0.23	0.372
	C3	-0.10	0.694	0.36	0.162	0.05	0.839
	CP3	-0.08	0.758	0.18	0.491	0.17	0.503
	CP5	0.22	0.399	0.04	0.891	-0.18	0.485
	AF4	0.03	0.898	0.15	0.566	0.38	0.131
	F1	-0.09	0.730	0.08	0.766	0.02	0.951
	F2	0.25	0.332	0.40	0.109	0.04	0.883
	F3	0.22	0.393	0.53	0.032	-0.10	0.694
	F4	-0.01	0.959	0.31	0.227	0.07	0.780
	F5	-0.08	0.766	0.49	0.048	0.03	0.913
	FC1	-0.08	0.766	0.21	0.410	0.27	0.294
	FC3	-0.28	0.272	0.28	0.268	0.09	0.723
	C3	-0.63	0.008	0.06	0.824	0.16	0.540
delta	C4	0.30	0.239	0.18	0.485	-0.13	0.612
	C5	-0.58	0.017	0.03	0.921	0.38	0.131
	C6	0.13	0.632	0.06	0.831	-0.28	0.281
	CP3	-0.30	0.243	0.10	0.708	0.27	0.299
	CP4	0.47	0.060	0.25	0.322	-0.26	0.308
	CP5	-0.49	0.048	0.23	0.377	0.21	0.426
	CP6	0.20	0.449	0.04	0.883	-0.21	0.415
	P3	0.16	0.528	0.20	0.432	0.41	0.106
	P5	0.20	0.438	0.34	0.178	0.41	0.100
	FP2	-0.10	0.715	-0.04	0.876	0.06	0.831
theta	AF3	0.17	0.509	0.15	0.572	-0.21	0.421
	AF4	0.11	0.687	0.21	0.410	0.17	0.503
	AF8	-0.04	0.891	0.00	1.000	0.20	0.438

F1	-0.06	0.831	-0.04	0.876	-0.34	0.188
F2	0.18	0.491	0.18	0.479	-0.32	0.212
F3	0.12	0.639	0.48	0.053	-0.14	0.579
F4	0.26	0.313	0.45	0.073	-0.06	0.817
F5	-0.11	0.680	0.44	0.082	0.03	0.898
F7	0.16	0.528	0.43	0.085	0.20	0.432
FC3	-0.17	0.509	0.24	0.352	-0.13	0.625
FT7	-0.01	0.981	0.03	0.913	0.46	0.068
C3	-0.40	0.109	0.15	0.553	0.02	0.943
C4	0.48	0.053	0.23	0.377	-0.09	0.730
CP3	-0.17	0.503	0.22	0.393	0.27	0.299
CP4	0.57	0.020	0.22	0.393	-0.39	0.120
CP5	-0.24	0.362	0.09	0.723	0.13	0.612
CP6	0.17	0.515	-0.12	0.646	-0.50	0.043
P3	0.31	0.220	0.15	0.553	0.09	0.744
P5	0.36	0.159	0.23	0.377	0.20	0.438
P6	0.31	0.223	-0.06	0.809	-0.24	0.357
P8	0.25	0.327	0.01	0.959	-0.21	0.415
O1	0.20	0.432	0.31	0.227	-0.20	0.443
O2	-0.13	0.625	0.07	0.787	0.05	0.839
